# Lectures on Diseases of the Chest

**Published:** 1845-09

**Authors:** J. R. Buck

**Affiliations:** Lecturer on the Theory and Practice of Medicine in the Louisville Summer School of Medicine; Louisville


### WESTERN JOURNAL

OF

### MEDICINE AND SURGERY.

SEPTEMBER, 1845.

Art. I.—*Lectures on Diseases of the Chest.* By J. R. Buck, M.D., Lecturer on the Theory and Practice of Medicine in the Louisville Summer School of Medicine.

LECTURE FOURTH.

PHTHISIS.—*(Concluded.)*

But when the disease is very extensive, issues are greatly preferable to any other mode of counter-irritation. They are less painful, do not heal up so readily, and discharge more freely. For establishing issues the Vienna paste is preferable to any other mode. It is made of equal parts of caustic potash, and quick-lime, rubbed up together. Placing a piece of adhesive plaster just beneath the clavicle, with a hole in it the size of a ten-cent piece, and covering the hole with the preparation mixed up into the consistence of paste with alcohol, the desired effect will be produced in from five to ten minutes; it must then be poulticed for ten or fifteen days until the eschar is removed; if it should manifest any tendency to heal up, two or three pieces of orris root kept in the issues by adhesive straps will be sufficient to keep up the discharge any length of time desirable. In this way all traces of inflammation are removed. The cough, however, may continue unabated, or may even be increased from the irritability of the part: it is just in this condition that cough mixtures are beneficial. They should never be given while there exists inflammation, as they serve by their anodyne property to lull the patient into a f.tal feeling of security, while the disease is stealthily but steadily progressing.

The following is the prescription that I generally use for allaying the cough:

℞ Morphiæ acetatis, gr.i.
Acaciæ mucilaginis, ℥vi.
Syrup. limonis, ℥ss.
Acid. hydrocyanic. medicinalis, gtt.x.
Mist.

Of this a table-spoonful three times a day, or when the cough is troublesome, should be given.

The next indication in this stage is the removal of tubercular matter from the lungs.

Can this be done? That tubercular matter is often removed from the lungs by expectoration and its place supplied by an earthy concretion, or by a semi-organized cartilaginous mass, is familiar to all pathologists. But whether it can be removed previous to softening by absorption, is a question, the affirmative of which is advocated by very few. There is certainly nothing in the composition of this matter which would seem to render it insusceptible to the action of the absorbent vessels; it differs but little from many articles of food, and is found occasionally in the blood, also in the lymphatic vessels; the thoracic duct has in some few instances been found distended with it. Cases are sometimes met with presenting all the symptoms and physical signs of tubercular matter in the lungs; these all disappear under treatment and never return; the presumption is that the matter has been absorbed; still, this fact is not conclusive, for it has already been mentioned that patients were often apparently restored to health, simply by the removal of the local inflammation or congestion produced by the tubercular matter, without at all disturbing the matter itself: but in such cases the disease will certainly return in the course of a year or two. Post-mortem examinations have thrown but little light upon this subject, from the little attention hitherto paid to physical diagnosis, by which alone the disease can be distinguished in the earlier stages of the deposit. In these cases, when the matter has been absorbed, either the lungs are not examined at all—the termination being obviously the consequence of some other disease—or the physician not being aware that they had ever been tuberculous, infers nothing from the absence of this matter.

Scrofulous tumors are often reduced or entirely removed by absorption previous to softening, and although I do not acknowledge the identity of this matter with tubercle, still so strong is the resemblance, that by many they are regarded as identical. Of the remedies supposed to possess the property of removing this deposit, the preparations of iodine have most celebrity. A combination of iodine with hydriodate of potash in solution, is a very popular remedy. It is objectionable from being exceedingly nauseous, and often disagreeing with the stomach after the first day or two. The iodide of iron which for a time was so popular in this stage of phthisis, but which from some cause has lost much of its popularity, I still regard, as a most valuable remedy, and as greatly facilitating the removal of tuberculous matter from the lungs. In addition to the tonic properties of this preparation indicated by the constitutional disease, the iodine acts specifically upon the absorbent vessels.

Mrs. T., æt. 32 years, had tubercles at the summit of the right lung, attended with a short, dry cough; in her family a strong predisposition to the disease existed; a brother was under treatment at the same time in the last stage of the disease. After congestion had been removed by local bleeding and efficient counter-irritation established, she was put upon syrup of the iodide of iron, a drachm to the ounce of syrup, dose a tea-spoonful three times a day. This is preferable to the aqueous solution, being more pleasant to the taste—less apt to disagree with the stomach, and beneficial to the slight irritation about the fauces and larynx so frequently present. Upon this treatment she gradually recovered, and at this time (nearly three years since she was discharged cured) there is no evidence of a return of the disease. I might refer to other cases identical in character. It should never be given when irritability or inflammation of the stomach exists, or when there is excitement in the system from any cause; this is doubtless the principal, if not the only cause of the unsatisfactory results which have attended its use; all excitement must be permanently reduced before it can be taken without injury.

Cod fish liver oil has, within the last few years, been highly recommended. In the only case in which I have seen it used, it produced such irritablity of the stomach after the second dose as to prevent its being continued. A highly respectable gentleman, one of the physicians to the Cincinnati hospital, informed me that he had given it a fair trial in a large number of cases, and that in no instance was any appreciable benefit perceived.

Naphtha.—This remedy was first brought to the notice of the profession by a Dr. Hastings. I shall give his own words: "The reasons which induced me to deviate from that line of medical treatment, which has so universally, and for so long a period, been in vogue, for that which I now submit to the profession, was the fatal termination of cases, whatever was the treatment adopted during an experience of upwards of twenty years. I was led to the conclusion from a careful survey of the chemical analysis of the tubercle, made by Rhenard, that it was defective, inasmuch as the composition of the animal matter which amounted to 98 parts in a hundred had not been investigated. From the greasy nature of tubercle, in its crude state, there did not exist the slightest doubt in my mind that carbon entered largely into its formation, and that its composition had a striking resemblance to fatty matter.

"In consequence of the loss of fat so remarkable in the earlier stages of consumption, I determined to employ those compound agents rich in carbon and hydrogen, which had not been previously used in medicine, not with the idea that they would make up the deficiency which the system had sustained in the progress of the disease, but that such a change would by that means be introduced into the constitution, as would act on the forces of the organism, at the point of departure from health, whether that took place in the stomach, blood, or elsewhere; that change tending to such an affinity in the elements within, that the carbon, oxygen, hydrogen, and nitrogen, instead of assisting in the formation of products which threaten, would tend to develop those materials only which are required for the perpetuation of health, and the prolongation of existence.

"I administered naphtha (pyro-acetic spirit) three times a day in doses of fifteen drops for an adult, mixed with a table-spoonful of water, which is proportionably decreased according as the patient approaches youth. After the second or third day I increase the dose by about one-fourth, regulating its increase or decrease according to the absence or presence of nausea, sickness, or any other untoward symptoms arising out of its use. As the disease advances increase the dose to forty and even fifty drops, and administer it four times a day instead of three times."

Chloride of sodium, or common salt, introduced into the treatment of phthisis by Dr. Latour, was tested by M. Louis in the Hotel Dieu for five months, and not in one single case did he perceive any advantage to result from its use. We conclude, therefore, that the three last named medicines are totally unworthy the reputation that they have enjoyed, and that it is extremely doubtful whether in any case of phthisis they are productive of benefit.

Mercury, although exerting a powerful influence upon the absorbent vessels, seems to increase the deposit of matter both in this disease and in scrofula: it is therefore rarely given. Whatever may be our treatment for the removal of tubercular matter from the lungs, we must not lose sight of the constitutional disease. The same treatment applicable to the first stage must be combined with it. The iodide of iron meets both indications.

Third stage.—Circumscribed pneumonitis, pleuritis, and bronchitis are constantly occurring in this stage. These must be treated by local bleeding, emetics, and counter-irritation. The only benefit ever derived from emetics in this disease is the relief of the secondary pneumonitis and bronchitis so frequently present. If by these means we can succeed in removing the inflammation, the disease will be arrested. One of the most troublesome symptoms connected with this stage is diarrhœa: if this be so profuse as to threaten the life of the individual, a combination of opium and acetate of lead, one grain of the former with five of the latter, every two or four hours, will most certainly and most speedily check it; but in less severe cases I have used the following prescription with the most satisfactory results:

℞ Sodæ muriatis,

Magnesiæ sulphatis, } ää ℥ss.

Acid. acetic. dilut.,

Aquæ menthæ, } ääOss.

Mist.

A table-spoonful three or four times a day will in a large majority of cases arrest the diarrhœa without constipating the bowels. The system in this stage requires to be constantly supported by a generous diet and by tonics. If there be much tightness in the chest with difficulty of expectoration, the vapor of tar should be inhaled: this can be most conveniently done by burning the tar in a tolerably close room in which the patient is confined. Inhalation of iodine and chlorine has also been used, but it is very questionable whether either of them possesses any virtue at all, nor are they entirely free from danger. I have seen most profuse hæmoptysis produced by iodine inhalations. Various external applications besides those mentioned are frequently used. Alcohol applied over the whole chest has been recently highly extolled, but with what justice I know not.

Change of climate is only beneficial in the very earliest stage of the tubercular deposit. When once the matter has begun to soften, no climate will compensate for the absence of friends and of those comforts to be met with at home alone. St. Augustine, in Florida, or Guines on the south side of the Island of Cuba, are perhaps the two points most favorable for tubercular patients.

Louisville, 1845.

